# Supplementary material for: Sesaminol Inhibits Adipogenesis by Suppressing Mitotic Clonal Expansion and Activating the Nrf2-ARE Pathway
Source: Nutrients. 2025 Oct 15;17(20):3242. doi: 10.3390/nu17203242 (PMC12567471; doi:10.3390/nu17203242)
Supplement: Supplementary file 1 [file nutrients-17-03242-s001.zip › Supplemental file 1.pdf]

Supplemental file 1

1. HPLC Analysis Conditions of purified sesaminol

Instrument: HITACHI Chromaster
Column:
  Guard Column: Wakosil-II 5C18HG (4.6 × 30 mm)
  Analytical Column: Wakosil-II 5C18HG (4.6 × 250 mm)
Mobile Phase:
  Solvent A: 10% acetonitrile + 0.1% trifluoroacetic acid (TFA)
  Solvent B: 80% acetonitrile + 0.1% trifluoroacetic acid (TFA)
Gradient Program:
  0–40 min: A 90% + B 10% → B 100%
  40–50 min: B 100% (column washing)
  50–60 min: A 90% + B 10% (re-equilibration)
Flow Rate: 0.80 mL/min
Column Temperature: 37° C (range: 36–38°C)
Sample Temperature: Room temperature
Detection Wavelength: 280 nm
Injection Volume: 10 µL

2. Representative Chromatogram of purified sesaminol

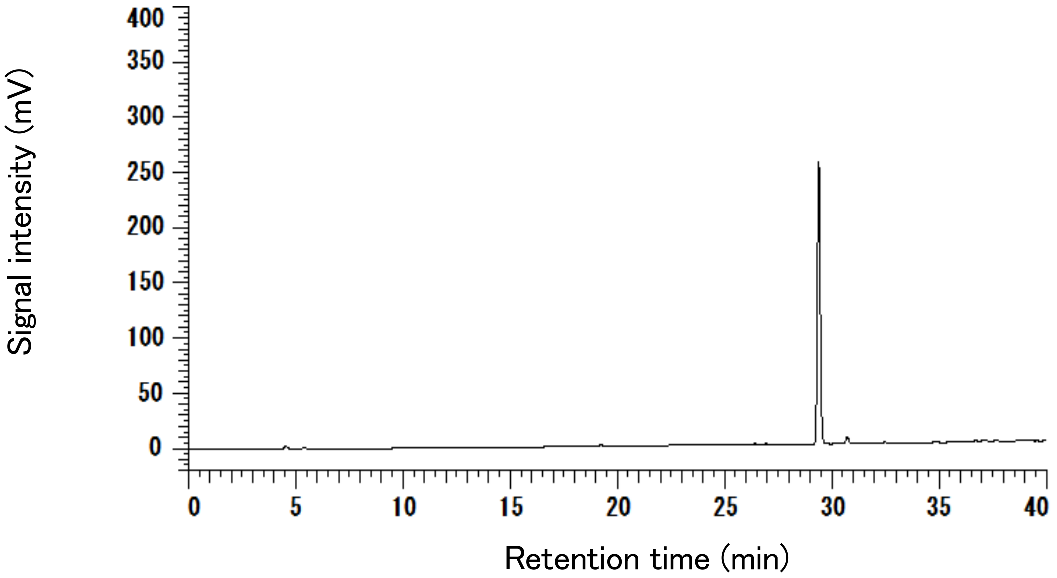

Representative HPLC chromatogram of purified sesaminol under the described conditions.
