# Supplementary material for: Sesaminol Inhibits Adipogenesis by Suppressing Mitotic Clonal Expansion and Activating the Nrf2-ARE Pathway
Source: Nutrients. 2025 Oct 15;17(20):3242. doi: 10.3390/nu17203242 (PMC12567471; doi:10.3390/nu17203242)
Supplement: Supplementary file 1 [file nutrients-17-03242-s001.zip › Supplemental file 2.pdf]

Supplemental file 2

C/EBPβ

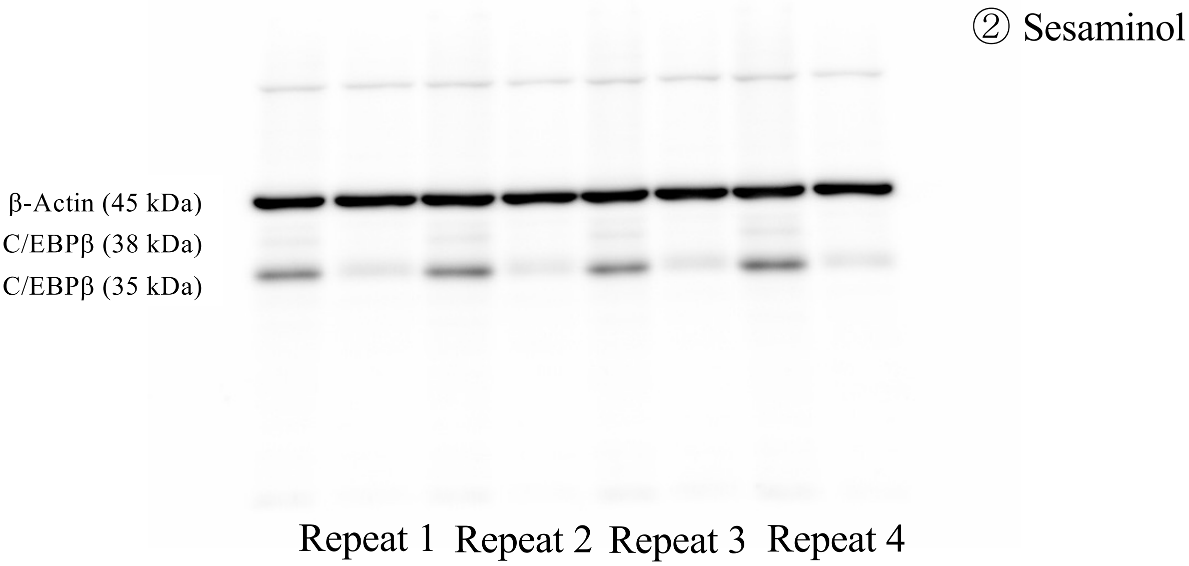

C/EBPα

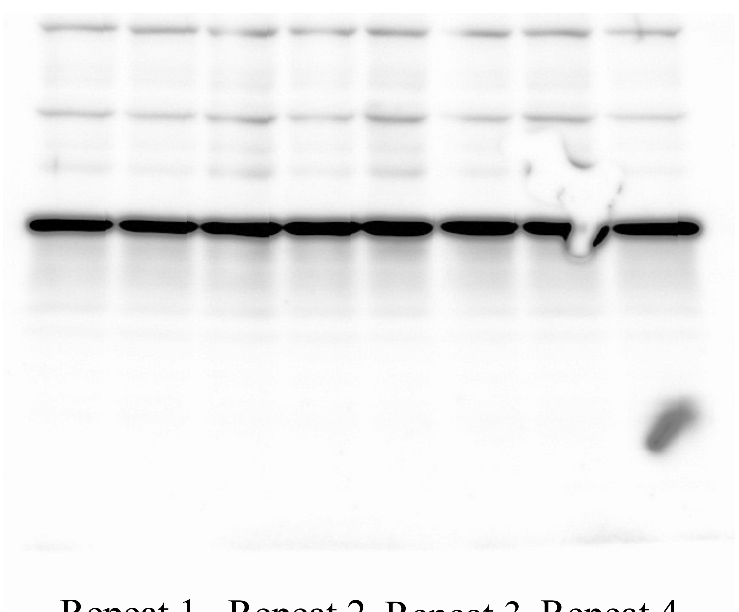

PPARγ

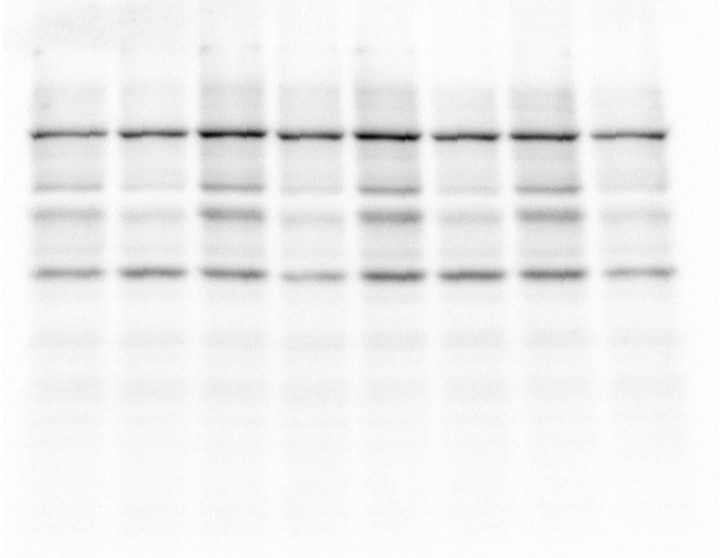

CDK2

Repeat 1 Repeat 2 Repeat 3 Repeat 4

①-1 ②-1 ①-2 ②-2 ①-3 ②-3 ①-4 ②-4

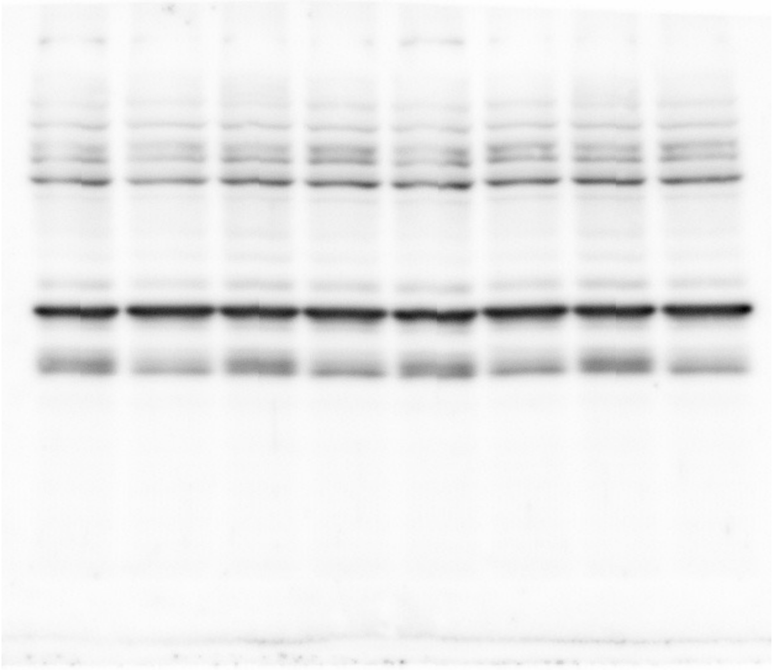

GAPDH (37 kDa)

CDK2 (33 kDa)

① Control
② Sesaminol

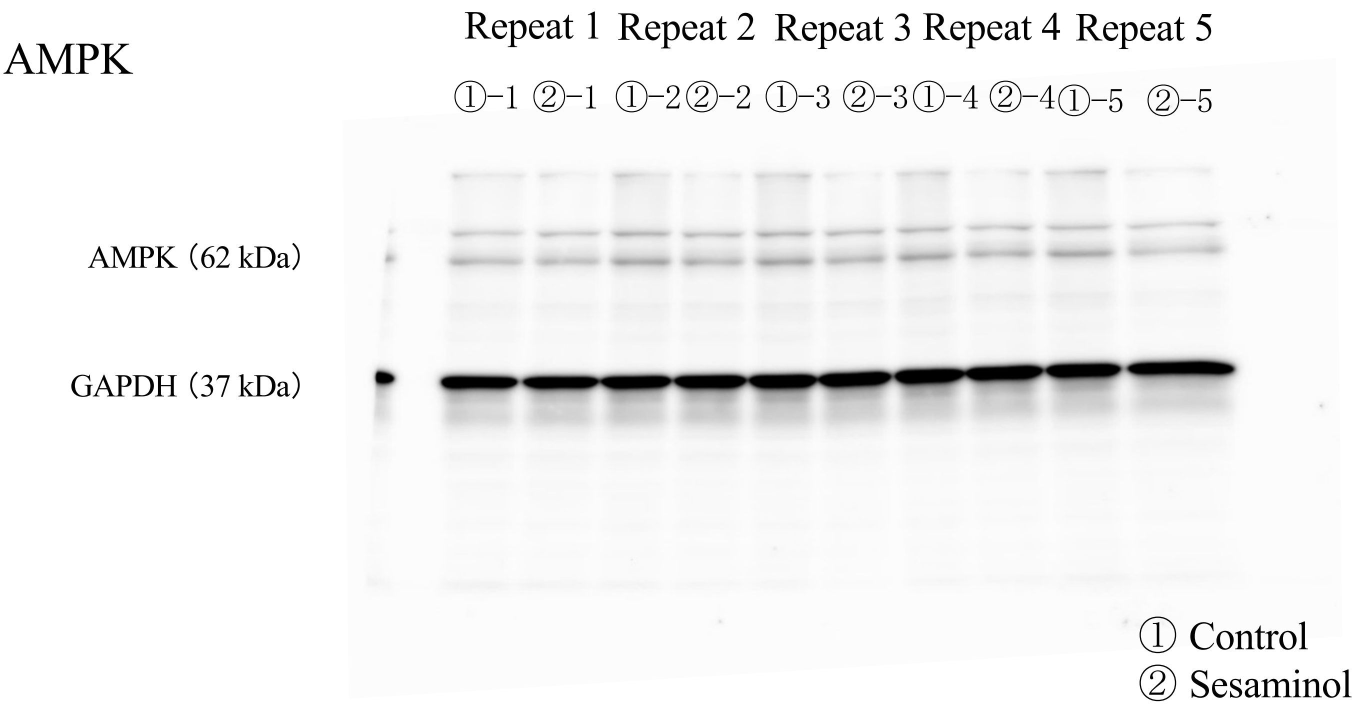

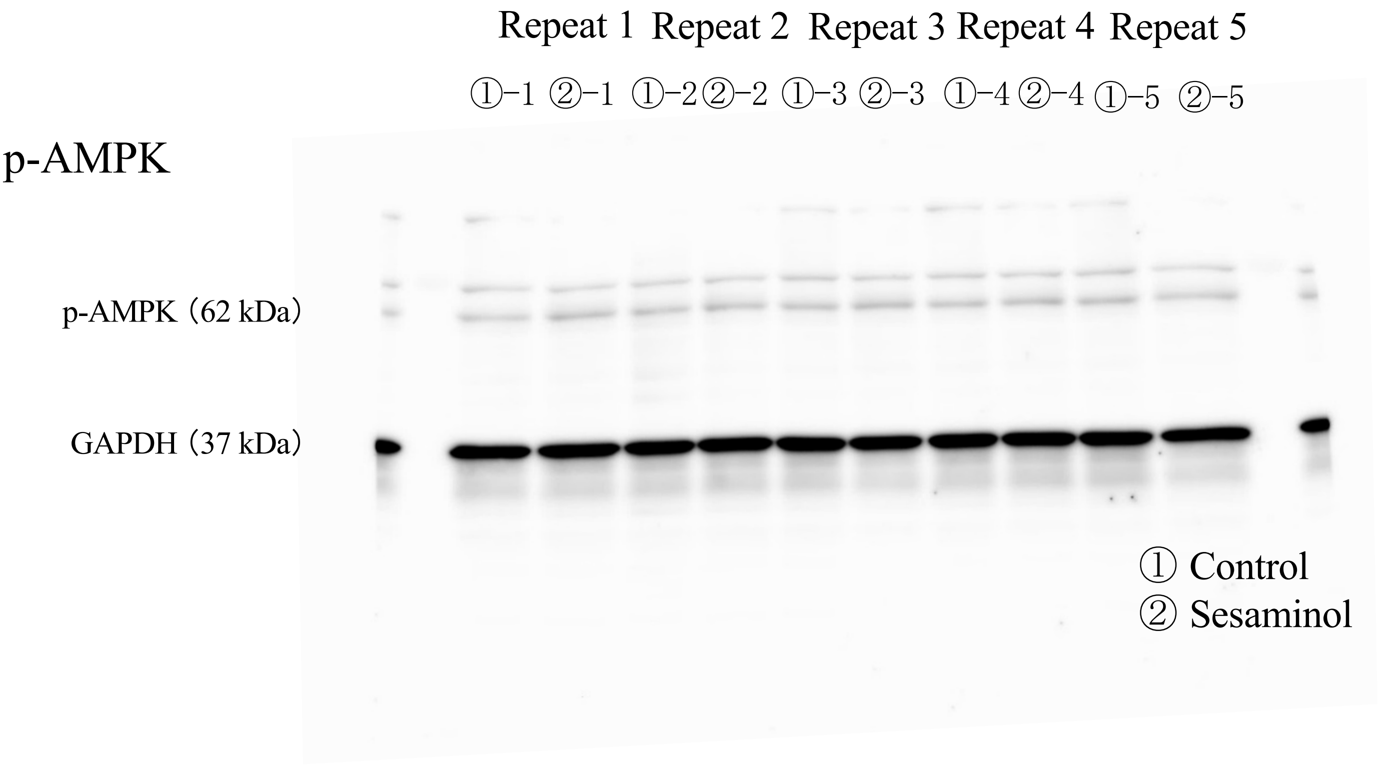

Protein expression levels of C/EBPβ, C/EBPα, PPARγ, , CDK2, AMPK or p-AMPK were measured repeatedly in the same gel using proteins obtained from cells cultured in four (or five) different dishes for two kinds of treatment, control① and sesaminol②.
